# Vaccine‐associated measles in an immunocompetent child

**DOI:** 10.1002/ccr3.1174

**Published:** 2017-09-12

**Authors:** Shawn B. Sood, Krishna Suthar, Kimberly Martin, Keith Mather

**Affiliations:** ^1^ Department of Pediatrics School of Community Medicine The University of Oklahoma Tulsa Oklahoma

**Keywords:** Immunization, measles, MMR, rash, vaccine

## Abstract

We present a rare case of vaccine‐associated measles infection in an immunocompetent, HIV‐negative patient in the United States. This case depicts the impressive rash our patient manifested and highlights the importance of reviewing public health interventions to determine epidemiological links in geographical areas with low incidence of measles.

## Graphical abstract

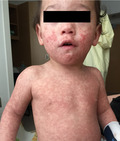

## Case Report

A previously healthy, 13‐month‐old immunocompetent male presented to our institution with a three‐day history of a diffuse maculopapular rash, fever, cough, and coryza. The patient developed his symptoms 9 days after receiving his first dose of the measles, mumps, and rubella (MMR) vaccine. He had no previous adverse reactions to immunizations. His past medical history was significant for allergic rhinitis for which he had been prescribed cetirizine, but the patient did not receive any medication for 10 days prior to his admission. The patient had no known sick contacts or any known contacts with a rash. The patient had no history of travel outside of Oklahoma, no barn yard animal contact, no ingestion of unpasteurized dairy products, and no consumption of undercooked meats. The patient was placed in a negative pressure room with strict droplet precautions.

**Figure 1:**
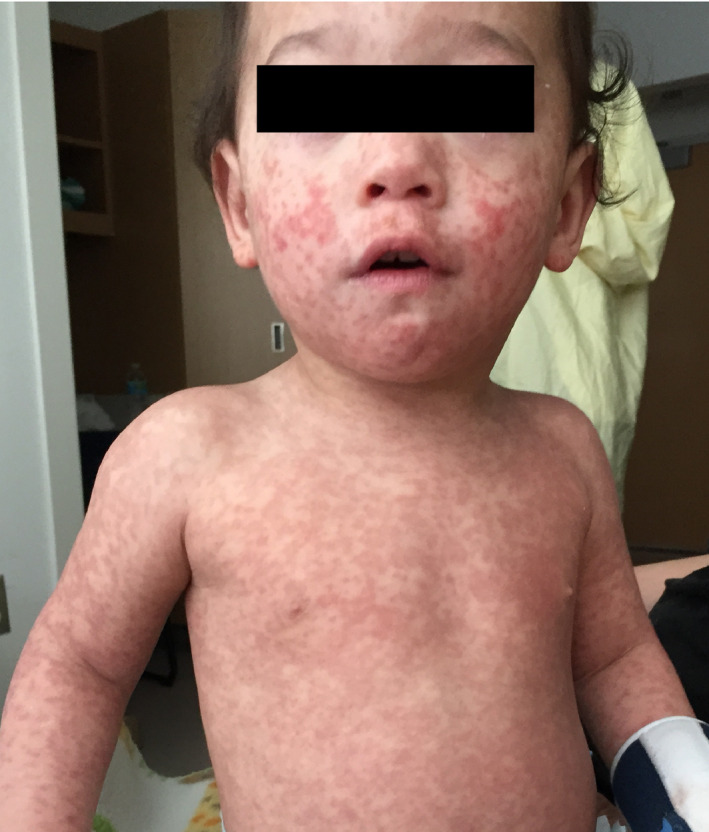
Full‐body maculopapular rash in our 13‐month‐old male patient.

**Figure 2:**
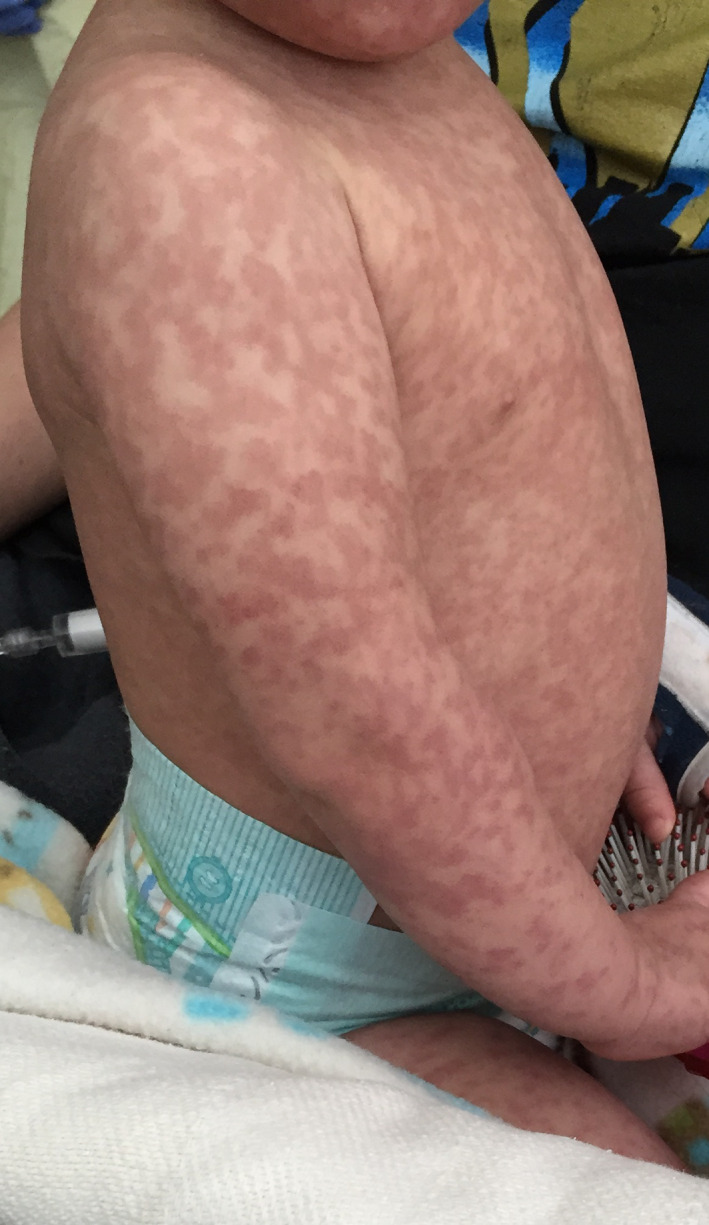
Full‐body maculopapular rash in our 13‐month‐old male patient.

His vital signs upon admission were stable. He was found to be positive for Respiratory Syncytial Virus (RSV) by nasopharyngeal PCR swab testing. On examination, the patient had a full‐body blanchable maculopapular rash (Figs 1 and 2).

All routine laboratory values were normal. A blood culture showed no growth after 5 days, and a urinalysis was negative for signs of infection. A nasopharyngeal PCR test for measles was obtained; testing for Human Immunodeficiency Virus (HIV), Cytomegalovirus, Ehrlichia, Epstein–Barr virus, Parvovirus B19, and Rocky Mountain spotted fever all resulted negative. The patient's rash and congestion resolved after 5 days of supportive care.

Reverse transcriptase‐polymerase chain reaction (RT‐PCR) testing for measles RNA from nasopharyngeal swab and measles‐specific IgM from serology both returned positive 2 weeks after the patient was discharged. The analysis was completed by the National Center for Immunization and Respiratory Diseases in Saint Paul, Minnesota. A positive measles IgM capture occurs if the mean optic density of the positive control serum reacted on viral antigen (P) subtracted from the mean optic density of the negative control serum reacted on viral antigen (N) is greater than or equal to 0.10 and the P to N ratio is greater than or equal to 3.00. If only one of these criteria is met, the results are equivocal. Our patient's P‐N was 2.103 and P/N was 30.406, both of which met criteria for a positive measles IgM – indicating an acute measles infection. Viral sequencing could not be performed as the sample collection was not sufficient. At the time of the patient's presentation, no known cases of wild‐type measles were reported in the state.

## Discussion

Symptoms of measles include fever, cough, coryza, and conjunctivitis. Within 5 days of symptoms, an exanthema described as an erythematous maculopapular eruption appears from the head and spreads caudally to cover the trunk and extremities 1, 2. Complications of measles include secondary bacterial pneumonia and encephalitis 2. Measles is highly contagious and has airborne transmissibility through respiratory droplets. No specific treatment is available beyond supportive care and Vitamin A 1.

The live‐attenuated vaccine is not given to pregnant patients or immunocompromised patients – for example patients with HIV, if CD4 counts are below 15% or 200 cells/*μ*L if age is >5 years 3. According to the Center for Disease Control and Prevention, an estimated 20 million people are infected with measles worldwide, making widespread vaccination a critical public health goal 3. In the United States, the measles vaccine is combined with the attenuated mumps and rubella vaccine viruses. The first dose is given at 12–15 months of age and the second between 4 and 6 years of age 3.

This vaccine induces both humoral and cellular immunity to provide long‐term protection from the wild‐type strain of measles 2. Shortly after vaccination, measles IgM and IgA are transiently positive in the blood and serum, respectively, but positive measles serum IgG persists for years, indicating immunity 2, 3. Similar to the wild‐type strain of measles, the vaccine strain induces measles virus‐specific CD4+ and CD8+ T lymphocytes and has both a stimulating and a considerably harmless, short‐lived suppressing effect on cell‐mediated immune responses 4. Adverse reactions after MMR vaccine administration are generally absent or mild. Low‐grade fever and mild rash occur in approximately 5% of children 2. Rarely, serious adverse events such as febrile seizures or anaphylaxis can occur 3.

We are aware of four cases of pediatric vaccine‐associated measles since 2001 – only one of which occurred in the United States 5, 6, 7, 8. The 13‐month patient from the United States had an indeterminate HIV status at time of infection 5. From the four other cases of pediatric vaccine‐associated measles and in the case of our patient, there was no known transmission of the virus to susceptible patients 5, 6, 7, 8. Our patient was of particular interest because he was immunocompetent, yet still manifested significant symptoms.

Laboratory analysis of the serum of patients with measles is essential to classify whether the strain of measles is wild‐type or vaccine‐associated, as both can have similar clinical presentations 5. While our patient did not have serologically confirmed vaccine‐associated measles, the suspicion was incredibly high based on both the patient's recent vaccine administration before his onset of symptoms and the epidemiologic data for measles in the state of Oklahoma showed no cases of wild‐type measles infection. It is possible that our patient's symptoms were due to his concomitant RSV infection. However, the timing of his symptoms after vaccine administration, his full‐body maculopapular rash, and the detection of measles virus by nasopharyngeal RT‐PCR were highly suggestive of vaccine‐associated measles infection.

The primary concern associated with vaccine‐associated measles is regarding the production of the vaccine batch. From a public health standpoint, it is critical to report possible cases of vaccine‐associated measles to the vaccine manufacturer and state authorities. One must assess the original vaccine batch for attenuation status of the live vaccine if a case of vaccine‐related measles occurs 5.

## Conclusion

Vaccine‐associated measles is a possible, but extremely rare event. We present a case of presumed vaccine‐associated measles in an immunocompetent, pediatric patient whose HIV status was confirmed to be negative at the time of presentation.

## Authorship

SBS: took responsibility for the accuracy of the case presentation, wrote and revised the manuscript, and provided direct care to the patient as Resident Physician. KS: contributed to the idea development and performed the literature search. KiM: Consultant Pediatric Infectious Disease Specialist provided direct care to the patient and made substantial contributions to revising the manuscript critically for important intellectual content. KeM: Pediatric Hospitalist Attending provided direct care to the patient and involved in conception of the work, critical revision of the article, and final approval of the version to be published. All authors contributed to the preparation, review, and submission of the manuscript to *Clinical Case Reports*.

## Conflict of Interest

None declared.
